# Peptide-Based Plasmon-Enhanced Spectroscopic Immunoassay to Detect Immunity Against Cytomegalovirus

**DOI:** 10.3390/bios15120817

**Published:** 2025-12-17

**Authors:** Aruna Chandra Singh, Clara Sidhoum, Hugo Payen, Divya Balakrishnan, Saulius Juodkazis, Thomas Østerbye, Sivashankar Krishnamoorthy

**Affiliations:** 1 Luxembourg Institute of Science and Technology (LIST), 41, rue du Brill, L-4422 Belvaux, Luxembourg; aruna.singh@imtek.uni-freiburg.de (A.C.S.); clsidhoum@gmail.com (C.S.); hugo.payen@list.lu (H.P.); divya.balakrishnan@list.lu (D.B.); 2 Optical Sciences Centre, Swinburne University of Technology, Hawthorn, VIC 3122, Australia; sjuodkazis@swin.edu.au; 3 Department of Immunology and Microbiology, University of Copenhagen, 22200 Copenhagen N, Denmark; thos@sund.ku.dk

**Keywords:** nanoplasmonic, metal-enhanced fluorescence, plasmonic nanoarray, anti-CMV, signal enhancement, surface plasmon resonance, fluorescence immunoassay

## Abstract

Sensors to monitor the immune status of an individual play a crucial role in understanding the acquired immunity or signs of a latent infection. Such sensors can be an effective tool to manage infection and to design treatment options in vulnerable populations. We demonstrate here highly sensitive detection of acquired immunity to Cytomegalovirus CMV by detection of anti-CMV antibodies using plasmon-enhanced fluorescence (PEF). The PEF sensors leverage plasmonic enhancement from a high density of intense electromagnetic hotspots in self-assembly-derived gold nanopillar arrays. Comparing PEF assays with assays on a planar surface plasmon resonance sensor shows the PEF sensors to be sensitive to a small fraction of the antibodies on the surface. The detection scheme deploys peptide monolayers with specific affinity to anti-CMV antibodies to capture them onto the sensor surfaces. The results of the assay on the PEF sensor reveal high promise for sensors with miniaturized sensing footprints, ease of spatial multiplexing, high sensitivity, and quick response times. The developments are readily applicable to a range of other diagnostic contexts where peptide–protein interactions and self-assembly-derived PEF sensors can be leveraged.

## 1. Introduction

Cytomegalovirus (CMV) is a widely prevalent herpesvirus that stays dormant in the body post-infection and can be transmitted via body fluids like saliva, breast milk, and blood. Once infected, the virus remains dormant in the body throughout one’s life and is activated when the host’s immunity weakens. The CMV is a concern in pregnant [1,2,3] or immunocompromised individuals, e.g., individuals with HIV and organ transplant recipients [4,5]. The role of CMV infections toward neurodegenerative diseases has long been hypothesized, with the cause–effect relationship still debated [6,7,8]. CMV infection can be detected either by detecting the virus or the antibody against the virus (anti-CMV antibodies). Anti-CMV antibodies are produced by the human immune system to fight the CMV infection. The detection of anti-CMV antibodies is the target of the diagnostic devices to determine an ongoing or past infection, to help design treatment options to manage the risk of infection in vulnerable individuals, and to prevent congenital infections that cause abnormalities in the fetus.

Anti-CMV detection is generally carried out using enzyme-linked immunosorbent assays (ELISAs) [9]. Polymerase chain reaction (PCR) or reverse transcriptase–quantitative PCR (RT-qPCR) is often used for diagnosis of congenital CMV infection [10]. Other reported alternatives include surface plasmon resonance (SPR) [11], quartz crystal microbalance (QCM) [12], Raman [13], and electrochemical sensing [14]. Amongst these, nanoplasmonic sensors based on plasmon-enhanced fluorescence (PEF) sensors are particularly interesting, as they bring high sensitivity, quick turnaround times, ease of multiplexing as microarrays, and ease of miniaturization into point-of-care configurations [15,16,17,18,19]. PEF sensors address the conventional limitations of ELISA [20,21] or PCR [22,23], including the long time to obtain results, cumbersome laboratory procedures, and high cost per measurement. They also address the limitations of SPR or QCM, with regard to their large measurement footprints, higher sample consumption, size that is less adapted to point of care configurations, and relatively high cost of ownership. However, advancement in PEF sensing requires better control over the size, shape, density, and distribution of electromagnetic hotspots to control both electromagnetic field distributions as well as how the analyte is distributed with respect to the hotspots. Furthermore, the ability to produce PEF sensors using quality-assured, scalable, and cost-effective approaches is important to deliver higher value and lower cost per measurement. We earlier showed gold nanoarrays of different geometries, including arrays of nanorods, nanodiscs, nanoparticle clusters, and nanopillars, and their application to plasmon-enhanced Raman and fluorescence sensing [24,25,26,27,28,29]. These arrays can be produced using self-assembly of block copolymer colloids, on full-wafer areas, and are adapted to further scalability using high-throughput techniques like nanoimprint lithography. We showed, for the first time, the use of self-assembly-derived high-density gold nanopillar arrays (Au NPAs) toward plasmon-enhanced fluorescence detection of anti-CMV antibodies using short-chain linear peptides as capture agents.

The use of peptides exhibiting specific affinity to capture anti-CMV [30,31,32] enables multiple advantages. Peptides serve as a synthetic, animal-free alternative for bioassays and support the EU directive 2010/63/EU that seeks to reduce the use of animals in research and development. Several reports have discussed the design, development, and application of peptides as receptors in bioassays targeting various disease targets [33,34,35,36,37,38,39,40,41]. The prospect of rational design, the ease of integration into sensors, along with stability under a wide range of conditions, and the ease of transportation and storage make peptides exhibiting characteristics that are both scientifically interesting as well as technologically promising. The smaller size of the peptides (a few amino acids) in comparison with an IgG (~1322–1534 amino acids) also reduces the overall thickness of the immunosandwich. This is particularly interesting for PEF assays, to achieve an optimally low distance between the fluorophore and the metal. This can also be favorably exploited in electrochemical sensors to achieve faster electron transfer rates with the underlying electrode. The present work, while discussing anti-CMV detection using self-assembly-derived PEF sensors, propound strategies that are readily extendible to other immunosensing scenarios. The ability to implement PEF sensors in microarray configurations further promises lower reagent consumption and a high multiplexing capability that are necessary to support the objectives of emerging personalized diagnostics and therapies.

## 2. Materials and Methods

Secondary goat anti-human IgG H&L (Cy5 ^®^) was purchased from Abcam (Cambridge, UK). MeO-PEG (7)–SH was purchased from Iris Biotech (Marktredwitz, Germany). 4-aminothiophenol (≥97%) was purchased from Sigma Aldrich (Saint Louis, MI, USA). Absolute ethanol (100%), acetone (99%), and 2-propanol (99%) were purchased from VWR Chemicals (Radnor, PA, USA). Polystyrene-*block*-poly(2-vinylpyridine) (PS-*b*-PVP) (197,000 g mol^−1^, block ratio of 1.07) was purchased from Polymer Source Inc. (Montreal, QC, Canada). Phosphate-buffered saline 10× buffer was purchased from Fischer Bioreagents (Waltham, MA, USA). The SPR sensing chips were purchased from Bionavis (Tampere, Finland). A peptide with a 16-amino acid sequence, Ac-GNSPWAPTAPLPGDM(EACA)C-NH_2_, and the anti-CMV antibody were provided by the Laboratory of Experimental Immunology, the Faculty of Health Sciences, the University of Copenhagen, Copenhagen, Denmark. A stock solution of the peptide was prepared by dissolving the lyophilized powder in DMSO and stored at −20 °C. The stock solution was diluted to 1 µg/mL in PBS prior to use.

The Si wafers used to fabricate PEF sensors, planar gold-coated silicon chips used as controls, and SPR gold sensors were all thoroughly cleaned using acetone and IPA, followed by UV/ozone exposure for 30 min before use. Gold-coated silicon chips were derived by coating gold thin films on Si <100> wafers using an MEB600S-HV electron beam evaporator (PLASSYS, Marolles-en-Hurepoix, France). Atomic force microscopy images of the templates used to create nanopillar arrays were acquired using an MFP-3D Infinity (Oxford Instruments, Oxfordshire, UK), operated in the tapping mode. Scanning electron microscopy (SEM) measurements of silicon and gold nanopillar arrays were acquired using Helios Nanolab 650 (Thermo Fisher Scientific, Waltham, MA, USA). SPR assays were performed on gold-coated sensor chips using multiparametric SPR Naali 220 A (Bionavis, Tempere, Finland) at 670 nm excitation, measuring the angular shift to the reflectance minimum. The SPR responses were calculated by the angular shift between the stable baselines in the buffer before and after the binding step.

The absolute reflection measurements on the Au NPAs were measured using a UV/visible spectrometer (Perkin Elmer Lambda 1050+, Springfield, IL, USA) in an integrated sphere configuration using a reflectance diffusive Teflon reference standard (WS-1, Ocean Optics, Miami, FL, USA). The electromagnetic field profiles for the Au NPAs were numerically simulated by using a three-dimensional finite-difference time domain (FDTD). The models used geometric parameters guided by experimental data. A built-in refractive index model was used for gold [42]. The background refractive index was set to be 1 (air). The whole structure was irradiated by a normally incident plane wave source in a wavelength range of 300–1000 nm. The anisotropic perfectly matched layer as the absorbing boundary condition was set at the *z* boundary, and periodic boundary conditions were set at the *x* and *y* boundaries of the simulation domain. An override mesh was used in the nanopillar arrays’ region with a smaller grid size than the default value for simulations. The grid sizes of 0.5 nm in the *x*- and *z*-directions and 1 nm in the *y*-direction were chosen to ensure numerical convergence.

Fluorescence measurements were performed using an Olympus BX53 system equipped with a 49913 ET filter set (Chroma Technology Corp, Bellows Falls, VT, USA) coupled to a QE Pro multichannel spectrometer (Ocean Optics, Orlando, FL, USA). The filter set enabled 633–640 nm excitation, and a long-pass emission filter was used to transmit light above 655 nm. The sharp edge due to the cut-on wavelength of the emission filter can be seen in the Cy5 spectrum. Fluorescence measurements were performed using a 50× objective with 0.95 NA with an exposure duration of 1 s. The spot size, taking 670 nm as the representative wavelength, was estimated at ~0.86 µm. Microspotting was performed using a Sciflex Arrayer S3 (Scienion, Berlin, Germany), with each droplet containing 320 pL of a 5 µg/mL solution of anti-CMV antibodies on the Au NPA substrate, pre-functionalized with the peptide and m-PEG thiol layers. Three drops were coated per feature across a square matrix to prepare a 5 × 5 array with a pitch of 500 µm. Following this, the whole surface was exposed to BSA and anti-human IgG. Fluorescence imaging of microarrays was performed using a GenePix 4400 A (Molecular Devices, San Jose, CA, USA).

## 3. Results

### 3.1. Gold Nanopillar Arrays as PEF Sensors

**Figure 1:**
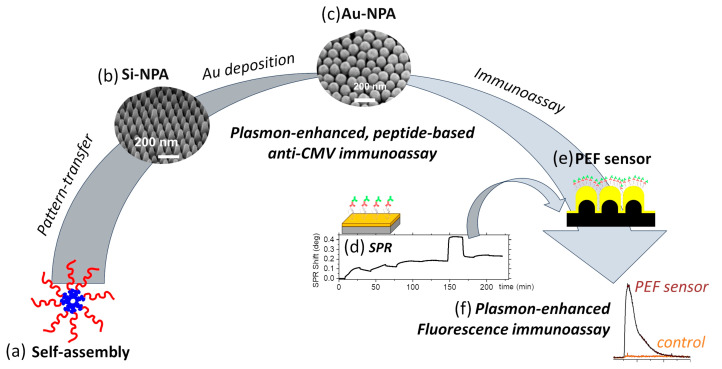
Workflow for fabrication and implementation of anti-CMV immunoassay, illustrating (**a**) molecular self-assembly to create (**b**) silicon nanopillar arrays by pattern-transfer, (**c**) gold nanopillar arrays upon metallization of silicon pillar arrays, (**d**) peptide-mediated anti-CMV immunoassay tested using surface-plasmon resonance and repeated on (**e**) plasmon-enhanced fluorescence sensor demonstrating (**f**) high fluorescence enhancement over planar controls.

Gold nanopillar arrays were prepared using self-assembly and pattern transfer of copolymer colloids using protocols that have been reported earlier (Figure 1a–c) [26]. Briefly, quasi-hexagonally ordered block copolymer colloids, with a pitch of 105.8 nm, were obtained by spin-coating a solution of PS-b-PVP from m-Xylene at 6000 rpm onto a 4″ Si wafer consisting of 25 nm SiO_2_ thermal oxide (Figure 1a,b). The organic pattern was transferred into underlying SiO_2_ and Si using a two-step process involving C_4_F_8_/CH_4_ and SF_6_/C_4_F_8_ plasmas to derive silicon nanopillars (Si-NPAs) as discussed in our earlier publications (Figure 1b) [26,43]. The pillar arrays can be attained on a full wafer level, with coefficients of variation in feature dimensions below 15%, with <10% variability in geometry across the wafer. The Si-NPAs were sputter-coated with 5 nm of Cr and 120 nm Au at a rate of 0.86 nm s^−1^ to form Au NPAs, with inter-pillar separations below 10 nm (Figure 1c). The ensuing Au NPAs exhibited a quasi-hexagonally periodic array with a pitch of 110 nm and featuring 12 billion pillars/cm^2^ (Figure 1c). The pillar arrays had inter-pillar gaps of ~8.4 nm that resulted in intense electromagnetic field enhancement at the inter-pillar gaps upon excitation with visible light(. These pillar arrays exhibited a hotspot density of ~4 × 10^9^/cm^2^, after factoring in the reduction in the number of hotspots caused by the fusion of the adjacent pillars [26]. These hotspots can be leveraged for highly sensitive plasmon-enhanced Raman or fluorescence spectroscopic detection of molecular analytes [26,44].

**Figure 2:**
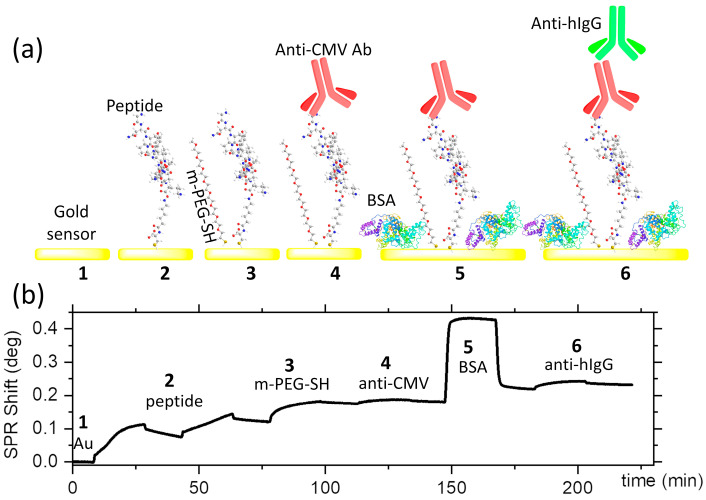
Anti-CMV immunoassay: (**a**) illustration of the steps of the peptide-based anti-CMV immunoassay and (**b**) the corresponding SPR response for each step.

**Figure 3:**
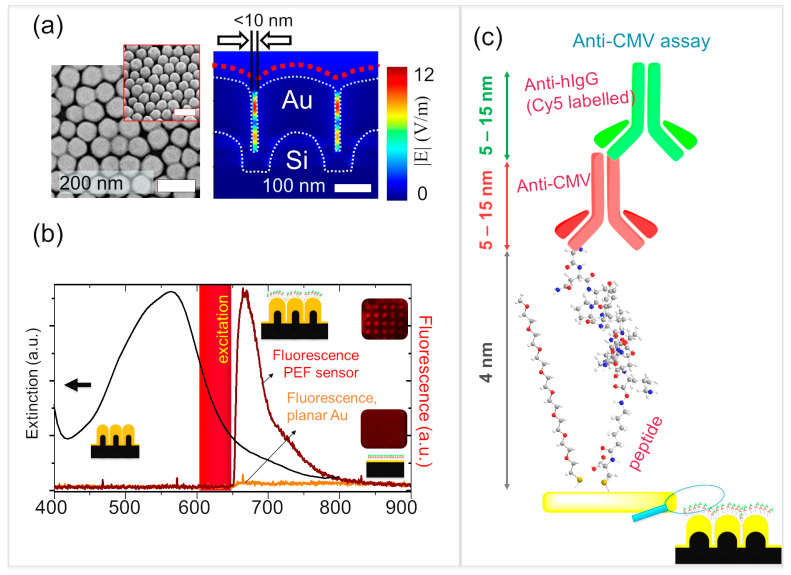
Plasmon-enhanced fluorescence sensor. (**a**) (**left**) Scanning electron microscopy measurements showing top view and (**left**, inset) 45° tilt view of Au NPAs (**right**); FDTD-simulated field maps showing intense electromagnetic field in the inter-pillar space. (**b**) (black curve) Extinction spectra of the Au NPA and the fluorescence spectrum of the endpoint of the immunoassay shown for the PEF sensor compared with the planar Au control. Fluorescence microarray shows distinct spots on the Au NPA, but not for the planar Au controls. The excitation band is shown for reference. (**c**) Illustration of the molecular arrangement at the endpoint of the immunoassay with indication of the corresponding molecular dimensions.

Here, we demonstrate plasmon-enhanced fluorescence-based rapid and sensitive detection of anti-CMV antibodies (Figure 1d–f). The PEF sensors adopted a sandwich immunoassay configuration (Figure 2a), where the capture of the anti-CMV antibody (the analyte) by the peptide-grafted sensor surface was transduced using the plasmon-enhanced fluorescence from the Cy5-conjugated detection antibody (Figure 1e,f). The assays relied on the peptide for specific capture of anti-CMV antibodies. The peptides were grafted onto the gold sensor surfaces of the SPR (Figure 2a) or the nanoplasmonic sensor (Figure 3) via cysteine residues at their N-terminal. Any non-specific binding sites in between the immobilized peptides were blocked using methoxy PEG thiols (m-PEG-SH) at high concentrations (Figure 2a). Following this, the anti-CMV antibody, if present, was captured from the medium. The surface was further treated with BSA before further exposure to Cy5-coated anti-human IgG for detection.

### 3.2. Measuring Peptide-Based Anti-CMV Immunoassays by SPR

The SPR assay on a planar gold sensor enabled rational interpretation of the outcomes of the PEF sensor by providing the density and kinetics of adsorption at each step and the presence of any non-specific binding. The SPR assays were carried out using sequential injection of reagents at a constant flow rate of 10 µL/min for a duration of 20 min, with 10 min of pre- and post-injection delays. The SPR responses for the different steps of the assay are shown in Figure 2b. In the first step, the gold SPR sensor was exposed to two consecutive injections of the peptide to maximize their grafting densities on the surface, which yielded a peptide density of 4.5 × 10^13^ molecules/cm^2^, or 0.46 molecules/nm^2^. This was followed by the injection of a 10 µM solution of methoxy PEG thiol, with a one-order-of-magnitude-higher concentration than the peptide solution (1 µM). The PEG thiols were used to block non-specific binding sites and were preferred instead of BSA to prevent the latter from sterically masking the peptides from being recognized by the anti-CMV antibodies. This is expected as the BSA has a molecular weight of 66.5 KDa, carrying 583 residues with typical geometric dimensions of 14 × 4 × 4 nm^3^ when dry. The peptides are, however, only 16 AA long, with an estimated dimension of 4 nm. The methoxy PEG (356.48 g/mol), in turn, has a typical length below 3 nm in fully stretched conformation. The reduction in non-specific binding is important to ensure the specificity of the sensor and to reduce the background, thereby assuring a high signal-to-noise ratio. The anti-CMV antibodies were flown at 5 µg/mL and were captured at a density of 4 ng/cm^2^, corresponding to 1.6 × 10^10^ molecules/cm^2^. BSA was subsequently injected to reduce any non-specific binding sites that may exist between the anti-CMV antibodies. At this stage, the addition of BSA was not a concern, as the peptide–antibody interactions had already occurred. In the final step, 10 µg/mL of Cy5-labeled anti-human IgG was used as a secondary antibody. The anti-human IgG is polyclonal, which would allow it to target multiple regions of the adsorbed anti-CMV. The SPR sensogram revealed an adsorption density of 13 ng/cm^2^, yielding a ratio of ~3 anti-human IgG per anti-CMV antibody. Negative controls showed a lack of binding of anti-human IgG in the absence of anti-CMV antibodies, as well as a lack of response when anti-mouse IgG was used in place of anti-human IgG.

### 3.3. PEF-Based Anti-CMV Immunoassays

The assays were subsequently repeated on the Au NPAs to realize a PEF sensor that profits from nanoplasmonic enhancement of fluorescence signals from the detection antibodies (Figure 3). The signal enhancement on the PEF sensors was investigated by performing the assay as shown in Figure 1a and comparing the fluorescence endpoint of the assay (Figure 3c) with that of the planar gold controls. The Au-NPA chips (or the planar controls) were exposed to the following steps, each separated by rinsing with 0.1 × PBS and blow drying with filtered N_2_: (a) peptide solution (200 μL) at 1 μg/mL for a duration of 90 min, (b) blocking non-specific binding sites using methoxy PEG thiol (200 µL) at 300 μM for 30 min, (c) anti-CMV antibodies (the analyte) (300 µL) at 5 µg/mL for 45 min, (d) Cy5-conjugated anti-human IgG as the detection antibody (300 µL) at 10 μg/mL for a duration of 1 h. The sample was washed and dried prior to fluorescence measurements. The end-point assay on the Au NPAs showed strong fluorescence enhancement, while the planar gold substrate barely showed any response under identical conditions (Figure 3b). Negative controls, as with SPR assays, showed a lack of fluorescence in the absence of anti-CMV antibodies. The PEF assay was independently implemented in a microarray configuration, where the spotting was performed with three drops of 320 pL of anti-CMV antibodies per feature, while performing the rest of the steps globally on the entire chip. The microarrays were clearly visible on the Au NPAs, while they were not seen in the planar gold controls treated under identical conditions (Figure 3b). Furthermore, further optimization of the microarray spotting conditions would enable higher density, reduced sample quantity, and improved spot uniformity.

## 4. Discussion

The success of PEF assays is dependent on both the degree of electromagnetic field enhancements at the hotspots as well as the ability of the fluorophore to leverage these enhancements. Furthermore, the spectral overlap between the molecular absorbance of the fluorophore and the plasmonic absorbance is important to ensure that the fluorophore and the localized surface plasmons can be excited simultaneously. It must, however, be noted that the correlation between the far-field spectra and the optical enhancements can be misleading for hotspot-dominated substrates. In the latter case, the nearfield enhancements at the hotspots do not correlate with the far-field plasmonic absorbance peak but have shown pronounced enhancements at wavelengths red-shifted from the plasmon resonance peak [45,46,47,48]. The localized surface plasmons of Au NPAs can be excited under normal incidence, unlike for continuous gold thin films, where the excitation of surface plasmon polaritons requires an angular incidence. Normal incidence allows for simpler measurement configurations and enables multiplexed read-out, as in the case of microarrays. Thus, the comparison in Figure 3b aims to bring out both the high fluorescence enhancement of the Au NPA as well as the low fluorescence intensities on the planar controls under equivalent conditions [49,50].

We showed earlier [26,44] that Au NPAs present high electromagnetic field enhancements in the inter-pillar region, maximizing at sub-10 nm pillar separations [26]. Our study also showed how the narrow inter-pillar hotspots restricted access to molecules larger than the inter-pillar gaps. The inability to access the inter-pillar hotspots was seen to lower the sensitivity in SERS assays, but delivered one-order-of-magnitude-lower limits of detection in plasmon-enhanced fluorescence assays. The plasmon-enhanced fluorescence assays can take advantage of the need to distance the fluorophore from the metal surface. Direct contact of the fluorophore with the gold surface would quench fluorescence due to rapid radiationless energy transfer to the metal. Several reports in the literature have pointed to the distance in the range of 5–90 nm between the fluorophore and the metal surface to be necessary to experience a plasmon-enhanced fluorescence enhancement [49,50,51]. In the present case, the immunoassay configuration would position the fluorophore at ~9–24 nm from the metal surface, estimating from the molecular dimensions of the peptide (4 nm), anti-CMV (~5–15 nm), and anti-human IgG (~5–15 nm), while factoring-in the possible different orientations that can be expected of the antibodies (Figure 3c). However, these distances are in solution and are expected to reduce further upon drying due to loss of hydration and due to changes in molecular conformation. In any case, it is reasonable to expect a distribution of the fluorophore across the thickness of the film, thus ensuring that at least a subset of the population of the fluorophores could contribute effectively to the enhancements.

**Figure 4:**
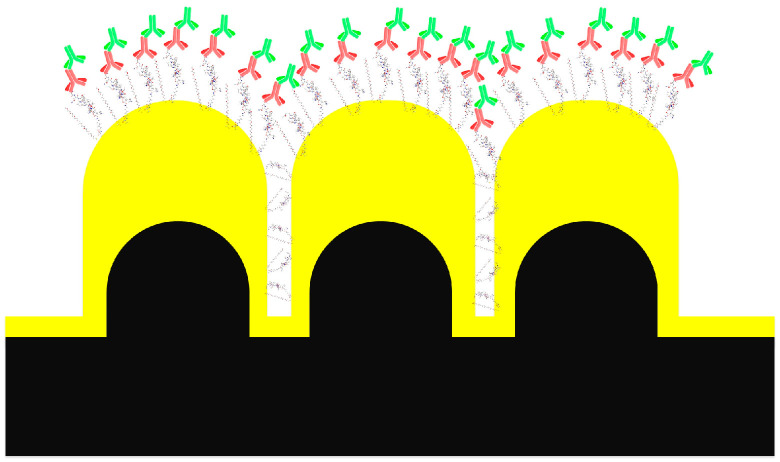
Illustration of steric hindrance on molecular distributions on the Au-NPA. The molecular construct, as shown in Figure 3c, is illustrated on top of the Au NPA. The larger size of the molecular construct in relation to the inter-pillar space is seen. The inter-pillar space may offer access to the peptide and m-PEG-SH, but it is not large enough to accommodate the antibodies (drawing not to scale).

While extrapolating the SPR measurements to the PEF assays, the impact of the geometry of the Au NPAs should be considered. The proximity of the pillars (~8.4 nm) in the Au NPAs would preclude the adsorption of the anti-CMV or the detection antibodies to the inter-pillar regions. The molecular interactions, as measured by the SPR, can, therefore, be extrapolated only to an unhindered gold surface at the very top of the pillars (Figure 4). Based on the geometry of the pillar arrays (pitch and diameter), the surface area at the top can be estimated to be equivalent to ~80% of a planar surface. This could reduce the surface densities of the Cy5-labeled anti-human IgG that contributes to the final signal. Factoring in the reduced densities, the PEF sensor can be expected to be sensitive to an estimated 416 molecules/µm^2^ of the anti-human IgG, and thereby, 130 molecules/µm^2^ of anti-CMV antibodies. This anti-CMV density represents only 0.4–1.6% of a monolayer of IgG, considering a typical monolayer of antibodies would constitute between 8000 and 32,000 molecules/µm^2^ (the range represents side-on to vertical antibody orientations). The high density of hotspots on the PEF sensors (~40/µm^2^) would mean a presence of approximately 10 molecules of the Cy5-labeled detection antibodies per hotspot, improving the chance of leveraging the hotspots toward enhancing the fluorescence signals. Although demonstrated as a PEF sensor, the same configuration could, in principle, be used for plasmon-enhanced Raman detection as well. In some cases, dual-mode sensing can provide unique advantages of complementary information through each of the sensing modalities employed [27,52].

The above estimates neglect the potential effects of the surface distribution at the top of the pillars, where the presence of curvatures could enhance the adsorption processes. We showed recently how nanotopographies could positively enhance the analyte capture efficiency on affinity sensors [53]. The curvature could also influence the distribution of molecules pre-disposing them to the inter-pillar regions [27,53]. In addition, the conformational freedom of the peptide chains could translate into enhancing the capture of anti-CMV antibodies [54,55,56]. While the results demonstrate a high signal enhancement on the PEF sensors in comparison with the bare gold surface, the contribution from the nanostructure variables via their impact on the molecular densities and distributions with respect to the inter-pillar electromagnetic hotspots cannot be excluded. Future work will shed light on the relative contribution of different variables to sensor performance by isolating and investigating them piecemeal. Techniques such as nano-QCM that use nanoarrays on a quartz crystal microbalance sensor, as we showed earlier [27,28,29,53,57], would be particularly well-suited in this respect. Self-assembly approaches, such as that used in this work, that can deliver well-defined nanoarrays on arbitrarily large areas with systematically variable nanostructure geometries at molecular-length scales have profound significance for nanoengineering of optical and bio-interfaces [26,43,58,59].

## 5. Conclusions

The use of a peptide-based plasmon-enhanced fluorescence immunoassay to detect antibodies against cytomegalovirus was demonstrated. The assay leverages a peptide with specific affinity to the anti-CMV antibodies as the capture layer. The confirmation of the peptide’s interaction with anti-CMV IgG, and the density of anti-CMV and Cy5-labeled anti-human IgG detection antibodies, was performed using surface plasmon resonance. The peptide-based immunoassay construct presents a size that predisposes the fluorophore to be positioned from the metal surface at distances that favor plasmonic fluorescence enhancement over fluorescence quenching. The ordered arrangement of the pillar arrays over large areas provides a high density of uniform electromagnetic hotspots that make it easier to build microarrays at any arbitrary position on the chip. The ordered arrangement also enables ease of modeling of the pillar array geometry to extrapolate the SPR outcomes to estimate the molecular densities on the PEF sensor. The fluorescence enhancements could be clearly differentiated over unstructured control surfaces via both microspectrometry and imaging. The self-assembly approach for fabricating the PEF sensor offers substantial scope to vary geometries to engineer electromagnetic field profiles around the nanopillars, as well as enhance the analyte’s leverage over the electromagnetic hotspots to maximize sensitivity. The results provide proof of concept of a PEF sensor that is promising for the combination of approach to fabrication, the choice of bioreceptor, and the sensitivity of the PEF sensor to anti-CMV antibodies with a surface density of < 2% of an antibody monolayer. The approach opens interesting avenues to advance miniaturized and multiplexed detection of biomarkers for a range of contexts of interest for diagnostics or the design of therapies. Future work should be pursued to validate and benchmark the performance of the sensor to detect and quantify analytes from body fluids like blood, saliva, and urine.

## Data Availability

The original contributions presented in this study are included in this article. Further inquiries can be directed to the corresponding author.

## Abbreviations

The following abbreviations were used in this manuscript:

PEF	Plasmon-enhanced fluorescence
SPR	Surface plasmon resonance
Au-NPA	Gold nanopillar array
